# CLEC3A-derived peptides exhibit broad-spectrum activity against *Candida auris* and clinically relevant pathogens

**DOI:** 10.3389/fcimb.2026.1756518

**Published:** 2026-02-23

**Authors:** Katinka Mies, Gabriele Hermes, Jens Beckers, Matthias Mörgelin, Michaela Simon, Tamara Rügamer, Jonathan Jantsch, Andreas R. Klatt, Thomas Streichert, Dzemal Elezagic

**Affiliations:** 1 Institute for Clinical Chemistry, Medical Faculty, University of Cologne, Cologne, Germany; 2 Colzyx AB , Lund, Sweden; 3 Institute of Laboratory Medicine and Microbiology, St. Marien Hospital, Amberg, Germany; 4 Institute for Medical Microbiology, Immunology and Hygiene, University Hospital Cologne and Faculty of Medicine, University of Cologne, Cologne, Germany; 5 Institute of Clinical Microbiology and Hygiene, University Hospital of Regensburg, Regensburg, Germany

**Keywords:** antimicrobial peptide (AMP), *Candida auris*, CLEC3A, ESKAPE, fungal biofilm

## Abstract

**Introduction:**

Antimicrobial resistance in bacterial and fungal pathogens poses a major threat to global health, with *Candida auris* recently classified by the WHO as a critical priority pathogen. Antimicrobial peptides (AMPs) have emerged as promising candidates due to their broad-spectrum activity and membrane-disruptive mechanisms.

**Methods:**

In this study, the antibacterial and antifungal efficacy of two CLEC3A-derived peptides, HT-47 and WRK-30, was evaluated in comparison to the reference AMP LL-37 and the drugs amphotericin B and penicillin/streptomycin using viable count assays, biofilm assays, and scanning and transmission electron microscopy.

**Results:**

HT-47 and WRK-30 showed antibacterial activity against the ESKAPE pathogens *K. pneumoniae* and *A. baumannii*, as well as antifungal effects against *C. albicans*, *C. neoformans*, and particularly *C. auris*, with MIC50 values comparable to or lower than amphotericin B. Both peptides significantly inhibited more potent *C. auris* biofilm formation, compared to amphotericin B. SEM and TEM revealed extensive membrane and subcellular damage in peptide-treated fungal cells.

**Conclusion:**

CLEC3A-derived peptides HT-47 and WRK-30 exhibit potent and comparable antibacterial and antifungal activity, highlighting their potential as therapeutic candidates for combating multidrug-resistant pathogens, including *C. auris*.

## Introduction

Antibiotics have revolutionised medicine in the fight against bacterial infections in recent years, but their overuse and misuse have led to the emergence of multidrug-resistant bacteria, posing a global problem (Tanwar et al., 2014; Ventola, 2015). Forecasts predict an increase in deaths from resistant infections from 700,000 (2014) to 10 million a year by 2050 (No time to wait: Securing the future from drug-resistant infections—Report to the Secretary-General of the United Nations; O´Neil, 2014). In 2019, bacterial resistance was directly responsible for 1.27 million deaths worldwide and contributed to a total of 4.95 million deaths (Murray et al., 2022).

A defined group of pathogens causes a substantial number of antimicrobial-resistant infections in clinical settings collectively referred to as the *ESKAPE* pathogens. This group comprises *Enterococcus faecium, Staphylococcus aureus, Klebsiella pneumoniae, Acinetobacter baumannii, Pseudomonas aeruginosa, and other members of the family Enterobacteriaceae*. These pathogens are of particular concern because they not only thrive in healthcare environments but also harbour a broad spectrum of intrinsic and acquired resistance mechanisms, making them major contributors to the global burden of drug-resistant infections (Anderson, 2005; Rice, 2008; Santajit and Indrawattana, 2016; Bongomin et al., 2017). *Klebsiella pneumoniae*, a gram-negative bacterium that naturally occurs, e.g., in the human gastrointestinal tract and can trigger pneumonia (e.g., community-acquired pneumonia (CAP)), especially in persons suffering from alcohol use disorder, diabetes mellitus, or during a hospital stay. Currently, K. pneumoniae is among the leading pathogens causing nosocomial infections, with a high global mortality rate (Podschun and Ullmann, 1998; Paczosa and Mecsas, 2016). *Acinetobacter baumannii* is a Gram-negative bacterium associated with global hospital-acquired infections; these infections occur particularly in critically ill patients in intensive care units (Fournier et al., 2006; Lin, 2014). Infections caused by this bacterium are varied and include pneumonia, urinary tract infections, meningitis, and skin/wound infections (Nasr, 2020). Carbapenems were long used as a preferred treatment for multidrug-resistant *A. baumannii* infections, but their frequent use led to the emergence of resistance. Extensive drug-resistant (XDR) *A. baumannii* shows resistance to at least three different antibiotics, while pan-drug-resistant (PDR) *A. baumannii* is additionally resistant to polymyxins and tigecycline (Nordmann and Poirel, 2019; Karakonstantis et al., 2020). Due to the rapid emergence of multidrug resistance to antibiotics, these bacteria exacerbate the growing global health issue (Boucher et al., 2009).

Not only can bacteria cause difficult-to-treat nosocomial infections, but fungi also pose a major problem due to the emergence of drug-resistant strains and limited antifungal therapeutic options (Wiederhold, 2017; Geddes-McAlister and Shapiro, 2019). *Candida albicans* is part of the healthy human microbiota and colonises the oral, gastrointestinal, and genital tract as a harmless commensal (Kennedy and Volz, 1985; Achkar and Fries, 2010; Ganguly and Mitchell, 2011). Malfunctions in the immune system or the environment can induce infections with *C. albicans*, ranging from superficial skin infections to life-threatening sepsis (Douglas, 2003). Immunosuppressed and cancer patients, as well as patients with medical implants, are particularly at risk (Kullberg and Oude Lashof, 2002; Pappas et al., 2018). *C. albicans* can form resistant biofilms on implanted material, which often cause the removal of the material and a high dose treatment with antimycotics, which results in health risk and economic costs (Kojic and Darouiche, 2004; Nobile and Johnson, 2015). Another critical fungal species is the basidiomycete Cryptococcus neoformans, which is ubiquitous in the environment and is often isolated from avian excreta or trees (Yamamoto et al., 1995; Lin, 2009). Due to the fungus´s natural occurrence in the environment, asymptomatic exposure via inhalation of spores is common. However, in immunocompromised individuals*, C. neoformans* can lead to pulmonary and systemic cryptococcosis, which often manifests as cryptococcal meningitis and results in high rates of fatalities, particularly among people with AIDS, where it accounts for an estimated 15-20% of AIDS-associated deaths globally (Park et al., 2009; Maziarz and Perfect, 2016; Tugume et al., 2023). The treatment of infections caused by this fungus is limited to a very few approved antifungal drugs. The widespread use of these drugs leads to rapid development of resistance and thus to infections that are increasingly difficult to treat (Lee et al., 2021). Additionally, *Candida auris*, a novel *Candida* species first reported in 2009 in Japan, has now already been reported on five continents (Satoh et al., 2009; Tsay et al., 2017). This opportunistic nosocomial pathogen can cause severe diseases in patients and is a major health care concern. The clinical relevance of *C. auris* is underscored by its recent classification by the World Health Organisation (WHO) as one of only five fungal pathogens of critical priority, highlighting the urgent need for novel therapeutic strategies (WHO fungal priority pathogens list to guide research, development and public health action, 2022). Unlike most other *Candida* species, it predominantly colonises the skin. As a result, the fungus can spread rapidly through skin-to-skin contact, potentially leading to candidemia, wound infections, or otitis (Heaney et al., 2020; Logan et al., 2020; Cristina et al., 2023). Notably, clinical isolates of *C. auris* exhibit substantial and in some cases untreatable resistance to all major antifungal classes, including azoles, amphotericin B, and echinocandins (Kilburn et al., 2022). One of the key factors contributing to the clinical complexity of *C. auris* is its capacity to form robust biofilm on medical surfaces, promoting environmental persistence and reducing susceptibility to antifungal treatment. These biofilm-associated communities are highly recalcitrant to eradication, often leading to persistent or recurrent infections despite conventional therapy (Ahmad and Alfouzan, 2021).

Given the global threat posed by multidrug-resistant bacteria and pathogenic fungi, there is a pressing need to explore alternative therapeutic strategies. Among the most promising are antimicrobial peptides (AMPs), small molecules naturally produced by bacteria, fungi, animals, and plants as a part of the innate immune defence (Lei et al., 2019). An advantage of AMPs is that they not only have antibacterial activity but also exhibit antifungal and antiviral activity, and, due to their small size, their production costs are low (Lei et al., 2019). Given the potential for hemolytic activity and the short half-life of natural AMPs in circulation, it is essential to design new AMPs or modify natural AMPs for therapeutic use (Moravej et al., 2018; Lei et al., 2019). A few AMPs are already in clinical trials. LL-37 is an AMP that has been extensively studied since its discovery in 2002 and is currently in a phase II clinical trial for hard-to-heal venous leg ulcers. LL-37 is the only cathelicidin (hCAP18)-derived antimicrobial peptide, which enhances wound healing by regulation of responses to inflammation (Ramanathan et al., 2002; Dürr et al., 2006; Grönberg et al., 2014). Melittin, an α-helical antimicrobial peptide and the primary component of bee venom, exhibits strong hemolytic activity toward human erythrocytes, which limits its systemic use. Nevertheless, due to its anti-inflammatory properties, melittin is used therapeutically, for example, in the treatment of inflammatory skin conditions (Lee and Bae, 2016; Dijksteel et al., 2021; Zhang et al., 2024). Another group of AMPs with potent and broad antimicrobial activity is CLEC3A-derived AMPs. C-type lectin superfamily A (CLEC3A) is a cartilage-specific member of the C-type lectin superfamily and consists of a signal peptide at the N-terminus (exon 1), an alpha-helical oligomerisation domain (exon 2), a carbohydrate recognition domain (CRD) (exon 3), and a 16 aa long region with eight positively charged amino acid residues (Neame et al., 1999).

Previous studies have shown that CLEC3A-derived peptides HT-16 and HT-47 exhibit antimicrobial activity against both Gram-positive and Gram-negative bacteria, including S. aureus, E. coli, and P. aeruginosa, as well as methicillin-resistant Staphylococcus *aureus* (MRSA) (Elezagic et al., 2019; Meinberger et al., 2023). Moreover, these peptides don´t exhibit toxic effects on primary human cartilage cells. Additionally, when coated onto titanium, a commonly used prosthetic material, the peptides significantly reduce bacterial adhesion (Elezagic et al., 2019). The peptides were further modified to improve their antimicrobial activity, biostability, and cytotoxicity (Meinberger et al., 2023). The peptide WRK-30, derived from HT-47, exhibited an even more potent antimicrobial activity profile and was less cytotoxic to murine fibroblasts than HT-47. Building on these findings, two CLEC3A-derived peptides (HT-47 and WRK-30) have emerged as promising candidates, demonstrating potent antimicrobial activity and *in vivo* efficacy in an implant-associated infection model. Such results highlight their therapeutic potential and raise the question of whether their activity extends to other ESKAPE pathogens and clinically relevant fungal species, thereby broadening their potential clinical applications.

Therefore, the study aims to comprehensively evaluate the antibacterial and antifungal activities of HT-47 and WRK-30 against clinically relevant bacterial and fungal pathogens, extending previous work by including additional ESKAPE pathogens and providing the first analysis of antifungal activity, mechanistic insights, and the biofilm-inhibiting potential of *C. auris*.

## Materials and methods

### Bacterial and fungal strains

Clinical isolates of *Acinetobacter baumannii*, *Klebsiella pneumoniae*, *Candida albicans*, *Cryptococcus neoformans*, and *Candida auris* were obtained from the strain repository of the Institute of Medical Microbiology at the University Hospital Cologne, which is according to ISO 15189, and were stored at -80 °C until use. The bacterial strains were cultivated in tryptic soy broth (TSB) at 37 °C with shaking at 200 rpm, or on TSB agarose plates at 37 °C. The fungal strains were cultivated in TSB at 37 °C with shaking at 200 rpm for liquid cultures. For solid cultures, C. albicans and C. auris were grown on TSB agar plates, while C. neoformans was cultured on Sabouraud agar plates at 37 °C.

### Peptide synthesis

HT-47, WRK-30, DK-29, and LL-37 were synthesised by Genosphere Biotechnologies (Clamart, France). They were provided in lyophilised powder form and exhibited a purity of more than 95%, as verified by liquid chromatography-MALDI-TOF mass spectrometry (LC-MS). All peptides were soluble in water to a concentration of 1 µg/µl (**Table 1**).

**Table 1:** Peptide names, molecular weights (kDa), and amino acid sequences (N- to C-terimuns) of CLEC3A-derived peptides (HT-47, WRK-30 and DK-29) and the reference peptide LL-37.

Peptide	kDa	Sequence (N- to C- terminus)
HT-47	5,542	HTSRLKARKHSKRRVRDKDDGLKTQIEKLWTEVNALKEIQALQTVCL
WRK-30	3,532	WRKHSKRRVRGGGLKTQIEKLWTEVNALKEI
DK-29	3,665	DKDGDLKTQIEKLWTEVNALKEIQALQTVCL
LL-37	4,5	LLGDFFRKSKEKIGKEFKRIVQRIKDFLRNLVPRTES

### Antimicrobial activity assay

Antimicrobial activity was assessed using a defined 2-hour killing assay to evaluate the effect of peptide exposure on bacterial and fungal viability. Bacteria or fungi were grown overnight in TSB at 37°C, in a shaking incubator at 200 rpm. The pathogen culture was grown to an optical density of approximately. 0.5 at 600 nm (OD_600_), after which the cells were harvested by centrifugation. The bacteria were diluted with tris-glucose buffer (TG buffer: 10 mM tris, 5 mM glucose, pH = 7.4) to a concentration of 2 x 10^6^ colony-forming units (CFU)/ml. Fungi were diluted with TG buffer to a concentration of 2 x 10^5^ CFU/ml. Afterwards, the pathogens were treated with the CLEC3A-derived peptides HT-47, WRK-30, and DK-29 in 96-well plates at 37 °C in a total volume of 100 µl per well. The peptides were incubated with the pathogens for 2 h, after which CFU counts were determined. LL-37, Pen./Strep., and amphotericin B (AmB) were included as positive controls. For the bacterial assay, the peptides were used at 5 dilutions from 5 µM to 0.3125 µM, and Pen./Strep. at 2 concentrations from 15 U/100 µl and 15 µg/100 µl to 0.9375 U/100 µl or µg/100 µl. Penicillin was supplied in biological activity units (U), which cannot be directly converted to a molar concentration. Streptomycin concentration corresponded to approximately 103 µM (based on streptomycin sulfate molecular weight). For the fungal assay, the peptide and AmB concentration ranges were 2.5 µM to 0.156 µM for C. albicans and C. neoformans, and 5 µM to 0.156 µM for *C. auris*. The pathogens were also cultured untreated as a growth control. After 2 h of incubation at 37°C, the pathogen suspensions were diluted using previously established dilution factors determined to yield colony counts suitable for quantification in the viable count assay. 25 µl of each approach was plated onto TSB agar plates (*K. pneumoniae, A. baumannii*, *C. neoformans, C. auris*) or Sabouraud agar plates (*C. albicans*) and incubated for 24h or 48h at 37 °C. The number of colonies was quantified the next day (24 h) for the bacteria,and fungi *K. pneumoniae and A. baumannii. C. auris* and after 48h for fungi *C. albicans and C. neoformans* using ImageJ cell counter. Fungal growth percentages were determined across peptide concentrations ranging from 0.15625 µM to 2.5 µM, normalized to the untreated control (100%). In parallel, colony counts were converted to CFU/ml and compared statistically with untreated controls.

### Statistical analysis

Statistical analysis of the results was performed using Prism 10.2.3 (403) (GraphPad, San Diego, CA, USA). Antimicrobial activity was analysed using one-way ANOVA followed by Dunnett´s multiple comparisons test, comparing each peptide-treated group to the untreated control. A significance level (α) of 0.05 was applied. P values are provided in the corresponding figure captions.

### MIC_50_ determination

The minimum inhibitory concentration (MIC_50_) that resulted in a 50% reduction in microbial growth was determined after performing viable count assays. Therefore, bacterial or fungal cultures were prepared as mentioned above and treated with the CLEC3A-derived peptides (HT-47, WRK-30, and DK-29), LL-37 as a positive control peptide, and a standard reference drug (Penicillin/Streptomycin for bacteria, amphotericin B for fungi) in a serial dilution (0.15625 – 5 µM). The MIC_50_ values were calculated using non-linear regression analysis in Excel. The number of replicates used for the calculation is the same as that of the viable count assays.

### Scanning electron microscopy

Overnight cultures of *Candida albicans*, *Candida auris*, and Cryptococcus neoformans were adjusted to a final cell density of approximately. 1 x 10^8^ cells/ml in TG buffer. Fungal suspensions (125 µl; ~1.25 x 10^7 cells) were then incubated with the respective peptides (10 µM) or amphotericin B as a control for 2h at 37°C. After incubation, samples were fixed by adding an equal volume of 8% formalin to reach a final formalin concentration of 4% per sample. Fungal suspensions (100 µl each) were applied onto 8 mm discs of freeze-dried collagen I matrix (Colzyx AB, Lund, Sweden) and incubated for 1 h at room temperature in a wet chamber. The specimens were then fixed in 2.5% glutaraldehyde prepared in 0.15 M sodium cacodylate buffer (pH 7,4), washed, and dehydrated through a graded ethanol series (50%, 70%, and 95% EtOH for 5 min each, followed by four washes in 100% EtOH for 5 min). Subsequently, the samples underwent critical-point drying using CO_2_ with absolute ethanol as the transitional solvent. Finally, they were mounted on aluminium stubs, sputter-coated with 20 nm palladium/gold, and imaged using a high-resolution dual-beam FEI Quanta 3D FEG scanning electron microscope at the Core Facility for Integrated Microscopy (CFIM), Panum Institute, University of Copenhagen.

### Transmission electron microscopy

A suspension of *C. auris* (2x10^8 cells/ml) was prepared in sterile PBS. Aliquots of 500 µl were incubated overnight at 37°C with either LL-37, HT-47, WRK-30, DK-29, or amphotericin B (AmB) at a final concentration of 10 µM. The following day, cells were collected by centrifugation at 4000 x g for 5 min, washed twice with 1 ml sterile PBS, and resuspended in 250 µl PBS. Samples were fixed by adding 250 µl of a fixation solution containing 2% glutaraldehyde, 2.5 sucrose, and 3 mM CaCl_2_ in 100 mM HEPES, followed by incubation for 30 min at room temperature and an additional 30 min at 4°C. After fixations, cells were washed in 0.1 mM HEPES buffer and pelleted at 1000 x g. Pellets were transported to the CECAD Imaging Facility at the University Hospital Cologne, where they were embedded in Epon resin and subsequently ultrathin-sectioned and imaged by TEM according to standard protocols.

### Biofilm formation assay

Biofilm formation was assessed using a modified crystal violet (CV) microtiter plate assay. C. auris overnight cultures in TSB were adjusted to OD600 = 0.1, and 90 µl were seeded into 96-well plates. Peptides were added as 10x stocks (final concentrations 2.5 -20 µM) with TSB as control, outer wells contained PBS to reduce edge effects. After overnight incubation at 37 °C, non-adherent cells were removed by washing with sterile water, biofilms were stained with 0.1% CV, and the bound dye was solubilised in 70% ethanol. Absorbance was calculated against untreated (100%) and medium-only (0%) controls.

### Ethical approval

The study utilised anonymised clinical isolates obtained from the hospital microbiology laboratory. Because no patient information or identifiable data were accessed, ethical approval was not necessary in accordance with institutional and national regulations.

## Results

### Quantification of antibacterial activity by viable count assay

**Figure 1:**
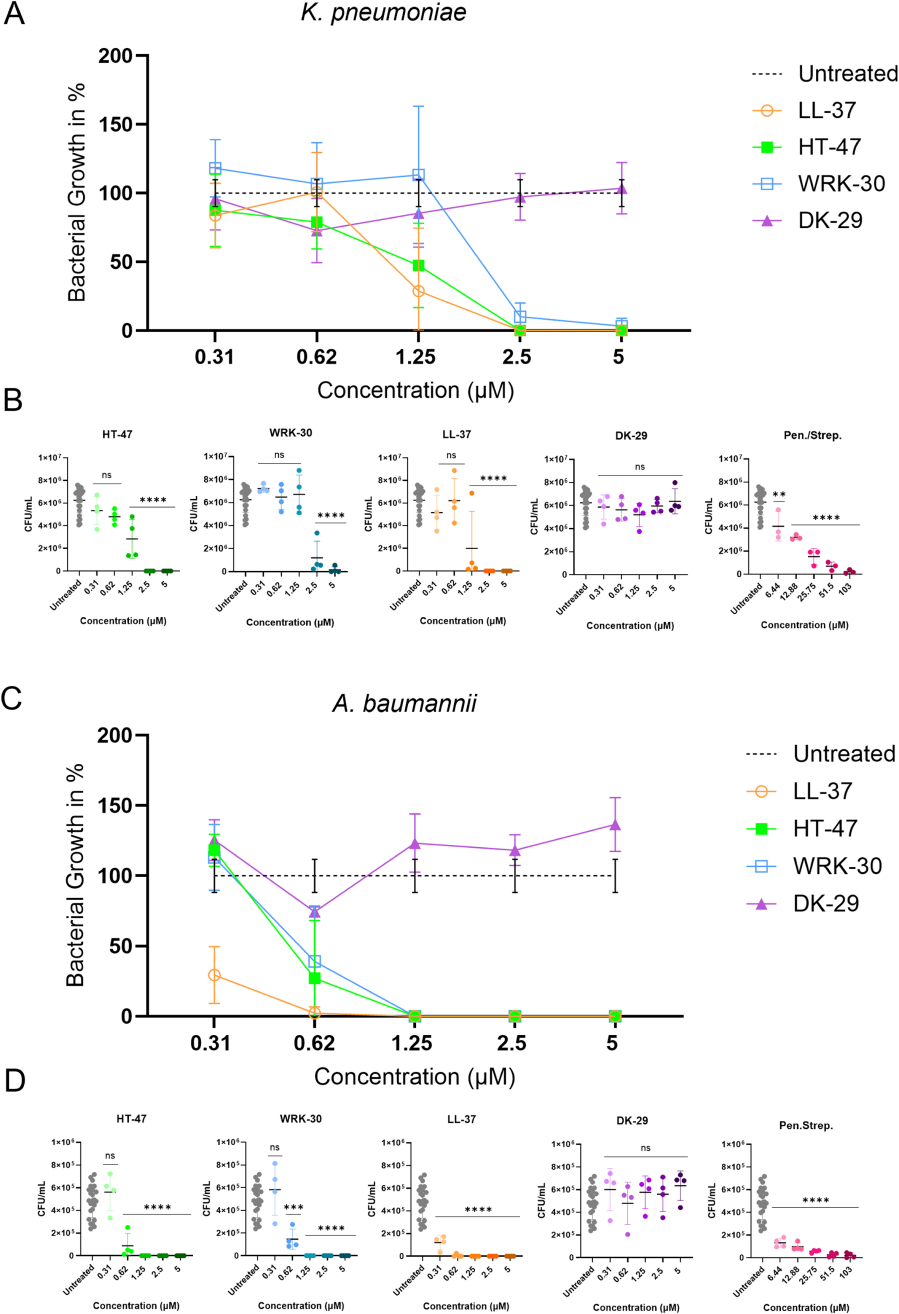
Antibacterial activity of CLEC3A-derived peptides against *K. pneumoniae* and *A. baumannii*. The antibacterial effects of HT-47, WRK-30 and the negative control DK-29 were compared with the reference peptide LL-37 and Penicillin/Streptomycin. Both *K. pneumoniae* **(A, B)** and *A. baumannii* **(C, D)** showed a significant reduction in viable counts following peptide treatment. Statistical analysis (one-way ANOVA with multiple comparison) revealed significant decrease in CFU/ml for *K. pneumoniae* with HT-47 (**** p < 0.0001), WRK-30 (**** p < 0.0001), LL-37 (**** p < 0.0001) and Pen./Strep. (** p = 0.0083; **** p < 0.0001) and for *A. baumannii* with HT-47 (**** p < 0.0001), WRK-30 (*** p = 0.0001, **** p < 0.0001), LL-37 (**** p < 0.0001) and Pen./Strep. (**** p > 0.0001) compared with untreated controls. For CFU/ml data, error bars represent the standard deviation (± SD) calculated from independent biological replicates. For percentage-normalised data, values were calculated individually for each biological replicate prior to averaging, and error bars represent the standard deviation (± SD) of these normalised values. No additional error propagation was applied. Penicillin was provided in units (U; not convertible to µM). Streptomycin was converted to µM.

The antibacterial activity of CLEC3A-derived peptides (HT-47, WRK-30, DK-29) against *K. pneumoniae* and *A. baumannii* was examined using viable count assays, in which bacterial growth was analysed as a percentage relative to untreated controls (100%) across peptide concentrations from 0.3125 µM to 5 µM. After incubation with the peptides, CFU/ml were quantified, and differences relative to the untreated control were determined (**Figure 1**). Incubation of *K. pneumoniae* with CLEC3A-derived peptides HT-47 and WRK-30 (**Figures 1A, B**) led to a significant reduction in bacterial load, starting at peptide concentrations of 1.25 µM and 2.5 µM, respectively, whereas LL-37 showed activity at 1.25 µM. In contrast, DK-29, as expected from previous work, had no antibacterial effect, whereas Pen./Strep. lead to a less significant reduction in bacterial growth at the lowest tested concentration (6.44 µM) (Elezagic et al., 2019). When incubated with *A. baumannii* (**Figure 1C, D**), both CLEC3A-derived peptides displayed significant reductions starting from 0.625 µM. LL-37 showed a significant reduction starting at 0.3125µM. DK-29 showed the same negative result as those for *K. pneumonia*e, whereas Pen./Strep. at a strong, significant reduction at its lowest tested concentration (6.44 µM).

### Quantification of antifungal activity by viable count assay

**Figure 2:**
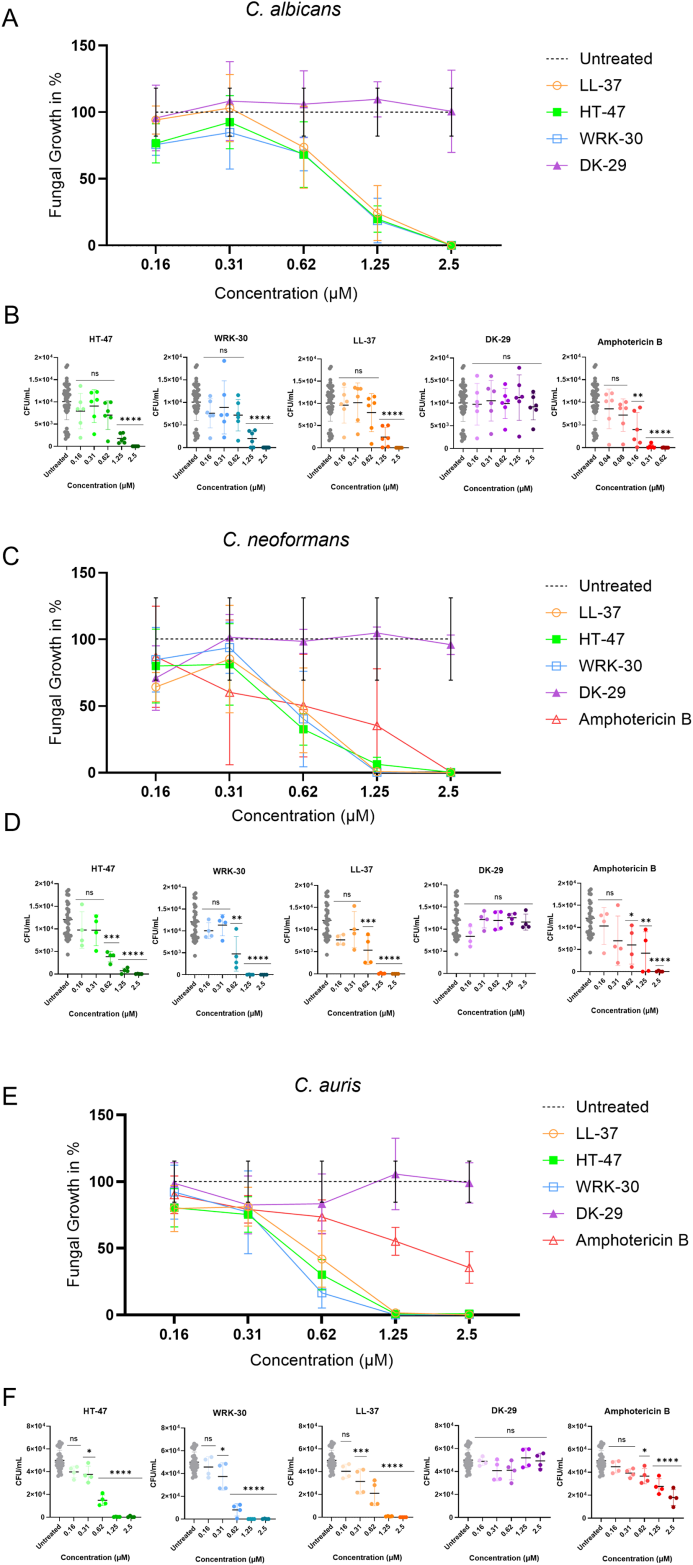
Antifungal activity of CLEC3A-derived peptides against *C. albicans*, *C. neoformans* and *C. auris*. The antifungal efficacy of HT-47, WRK-30 and the negative control peptide DK-29 was compared with LL-37 and amphotericin B (AmB). All three fungal species showed concentration-dependent growth reduction following peptide treatment. Statistical analysis (one-way ANOVA with multiple comparison) revealed significant decreases in CFU/ml for *C. albicans* **(A, B)** HT-47 (**** p < 0.0001), WRK-30 (**** p < 0.0001), LL-37 (*** p = 0.0001, **** p < 0.0001) and AmB (** p = 0.0019, **** p < 0.0001), for *C. neoformans* **(C, D)** HT-47 (*** p = 0.0004, **** p < 0.0001), WRK-30 (** p = 0.0015, **** p < 0.0001), AmB (* p = 0.0384, ** p = 0.0035, **** p < 0.0001) and LL-37 (** p = 0.0045, **** p < 0.0001) and for *C. auris* **(E, F)** HT-47 (* = 0.0198, **** p < 0.0001), WRK-30 (* p = 0.0347, **** p < 0.0001), LL-37 (*** p = 0.0008, **** p < 0.0001) and AmB (* p = 0.0162, **** p < 0.0001) compared with untreated controls. For CFU/ml data, error bars represent the standard deviation (± SD) calculated from independent biological replicates. For percentage-normalised data, values were calculated individually for each biological replicate prior to averaging, and error bars represent the standard deviation (± SD) of these normalised values. No additional error propagation was applied.

The antifungal activity of CLEC3A-derived peptides against *Candida albicans*, *Cryptococcus neoformans*, and *Candida auris* was also evaluated using viable-count assays (**Figure 2**). When incubated with *C. albicans* (**Figure 2A, B**), significant reductions in fungal growth were observed starting with a concentration of 1,25 µM for the CLEC3A-derived peptides HT-47 and WRK-30, as well as LL-37. At the same time, AmB led to antifungal activity at a concentration of 0.16 µM. As expected, DK-29 had no antifungal effect. For *C. neoformans* (**Figure 2C, D**), significant reductions were observed with a concentration of 0.625 µM for HT-47, WRK-30, LL-37, and 0.16 µM for AmB, whereas DK-29 again showed no decrease in fungal load. When incubated with *C. auris* (**Figure 2E, F**), HT-47, WRK-30, and LL-37 reduced fungal growth significantly, starting at a concentration of 0.31 µM, whereas a concentration of 0.16µM of AmB was needed for the antifungal activity, with DK-29 remaining inactive. 

### Determination of MIC_50_ values for bacterial and fungal pathogens

The MIC_50_ values for the CLEC3A-derived peptides (HT-47, WRK-30, and DK-29), the antimicrobial peptide LL-37, as well as the control drugs amphotericin B (for fungi) and penicillin/streptomycin (for bacteria), were determined based on the results of viable count assays (**Table 2**). For *K. pneumoniae*, MIC_50_ values ranged from 1.14 µM (LL-37) to 9.74 µM (amphotericin B. In *A. baumannii*, the MIC_50_ values ranged from 0.33 µM (LL-37) to 0.63 µM (WRK-30). Among the fungal pathogens, the MIC_50_ values for *C. albicans* ranged from 0.1 µM (amphotericin B) to 0.87 µM (WRK-30), and for *C. neoformans* from 0.21 µM (amphotericin B) to 0.59 µM (WRK-30). The MIC_50_ values for *C. auris* ranged from 0.39 µM for amphotericin B to 0.60 µM for WRK-30. Across most tested pathogens, MIC_50_ values for the CLEC3A-derived peptides did not differ significantly from those of LL-37 or conventional antimicrobial controls, indicating comparable antimicrobial potency.

**Table 2:** MIC_50_ values (in µM), determined by viable count assay, of selected antimicrobial peptides (LL-37, HT-47 and WRK-30) and controls (amphotericin (AmB), Penicillin/Streptomycin (P./S.)) against various clinically relevant pathogens, including Gram-negative bacteria (*K. pneumoniae, A. baumannii*) and fungal species (*C. albicans, C. neoformans, C. auris*): Values represent the mean ± standard deviation from at least four independent biological replicates. “n.a.” indicates that no MIC_50_ could be determined.

Pathogen	*K. pneumoniae*	*A. baumannii*	*C. albicans*	*C. neoformans*	*C. auris*
LL-37 (µM)	1.14 ± 0.49	0.33^x^	0.85 ± 0.29	0.57 ± 0.31	0.60 ± 0.16
AmB (µM)	n.a.	n.a.	0.1 ± 0.08	0.21 ± 0.17	0.39 ± 0,13
P./S. (µM)	9.74 ± 2,8	< 6.44^xx^	n.a.	n.a.	n.a.
HT-47 (µM)	1.24 ± 0.47	0.61 ± 0.19	0.78 ± 0.26	0.46 ± 0.2	0.49 ± 0.091
WRK-30 (µM)	2.22 ± 0.78	0.63 ± 0.20	0.87 ± 0.15	0.59 ± 0.22	0.42 ± 0.12

Statistical analysis was performed using an ordinary one-way ANOVA with multiple comparisons, in which the mean MIC50 value of each group was compared with those of all other groups. ^x^The MIC_50_ value for LL-37 and Pen./Strep. against *A. baumannii* is based on a single replicate, as in all other replicates, bacterial survival was lower than 50%, which did not allow for proper MIC_50_ determination. ^xx^ Pen./Strep. did not reach the MIC_50_ threshold against *A. baumannii* at any concentration tested, indicating that the MIC_50_ lies below 6.44 µM.

### Impact of CLEC3A-derived peptides on *C. auris* biofilm formation

**Figure 3:**
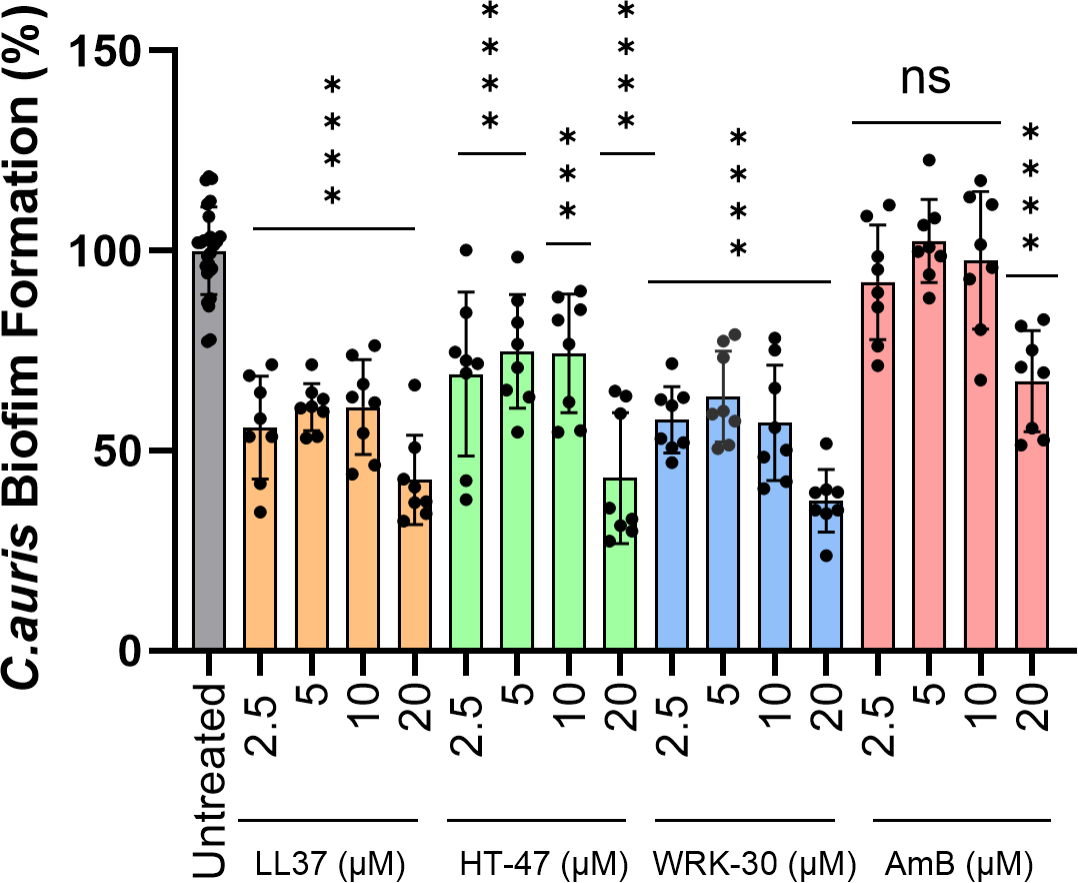
Inhibition of biofilm formation. The effects of the CLEC3A-derived peptides HT-47 and WRK-30, the antimicrobial peptide LL-37, and the antifungal drug amphotericin B (AmB) on *C. auris* biofilm formation were assessed using a crystal violet (CV) microtiter plate assay. Biofilms were grown overnight under static conditions in the presence of increasing concentrations of peptides or AmB (2.5 to 20 µM). After washing and CV staining, biofilm biomass was quantified by absorbance measurement. Untreated controls were defined as 100% biofilm formation (grey). All peptides, as well as AmB, showed concentration-dependent reduction in biofilm biomass. Statistical analysis (one-way ANOVA with multiple comparisons) confirmed significant inhibition by HT-47 (*** p = 0.0002 and 0.0001, **** p = < 0.0001), WRK-30 (**** p = < 0.0001), LL-37 (**** p = < 0.0001), and AmB (**** p = < 0.0001) compared to untreated controls. Data represent three independent experiments (n=3) for peptides and AmB with at least duplicates (untreated controls n=25).

The CLEC3A-derived peptides HT-47 and WRK-30 were evaluated for their ability to inhibit biofilm formation in *C. auris* with LL-37 as a reference AMP and amphotericin B (AmB) as an antifungal agent. Biofilm formation of untreated cells was set to 100%, and the relative biofilm formation of treated cells was calculated accordingly. All treatments demonstrated a concentration-dependent reduction in *C. auris* biofilm formation. HT-47 and WRK-30 reduced biofilm formation by 27% and 45%, respectively, at a peptide concentration of 2.5 µM. At the highest peptide concentration used in our study (20 µM), HT-47 reduced biofilm formation by 57%, and WRK-30 by 64%. LL-37 achieved a comparable effect to WRK-30. In contrast, AmB showed no significant inhibition at lower concentrations and reduced biofilm formation by only 36% at 20 µM, compared with the untreated control (**Figure 3**).

### Visualization of peptide-induced fungal damage by transmission electron microscopy

**Figure 4:**
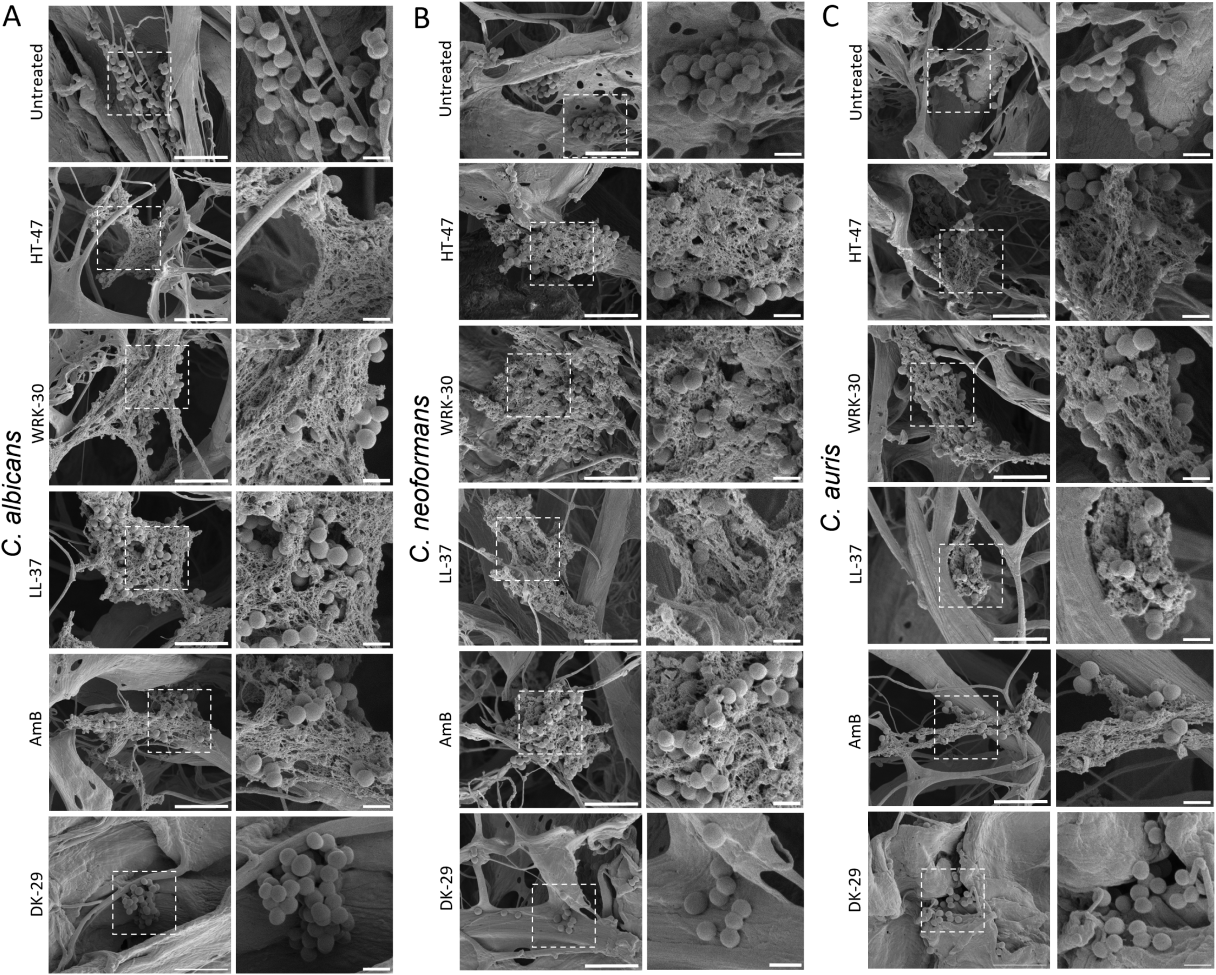
Scanning electron microscopy of *fungi* after peptide treatment. *C. albicans, C. neoformans, and C. auris were incubated with 10 µM of HT-47, WRK-30, DK-29*, and LL-37 as well as amphotericin B (AmB) or left untreated. **(A)** shows representative SEM images of C. albicans under the indicated conditions. **(B)** shows representative SEM images of C. neoformans and **(C)** representative images of *C.auris*. For each treatment, an overview image (left) is displayed alongside a corresponding higher-magnification zoom-in (right), with the corresponding region highlighted in the overview by a white box. Scale bars: 5 µm (overview images) and 1 µm (zoom-ins). In all samples, fungal cells appear as spherical structures attached to the underlying collagen I matrix, which served as a support during sample preparation.

To investigate the morphological effects of CLEC3A-derived peptide on fungi, scanning electron microscopy (SEM) was performed on *C. albicans*, *C. neoformans*, and C. auris after incubation with 10 µM of HT-47, WRK-30, or DK-29. In untreated C. albicans controls (**Figure 4A**), cells exhibited the typical spherical morphology, with smooth, intact surfaces and dense attachment to the collagen matrix. In contrast, treatment with HT-47 or WRK-30 resulted in extensive cell destruction, as evidenced by large areas of fibrous debris in which only a few intact fungal cells remained. A similar pattern was observed after treatment with LL-37 and AmB, in which widespread cell lysis led to the accumulation of fragmented material. In DK-29-treated samples, however, cells retained their typical morphology, resembling the untreated controls. This was observed for *C. neoformans* (**Figure 4B**), as well as *C. auris* (**Figure 4C**) Visualisation of peptide-induced fungal damage by transmission electron microscopy.

**Figure 5:**
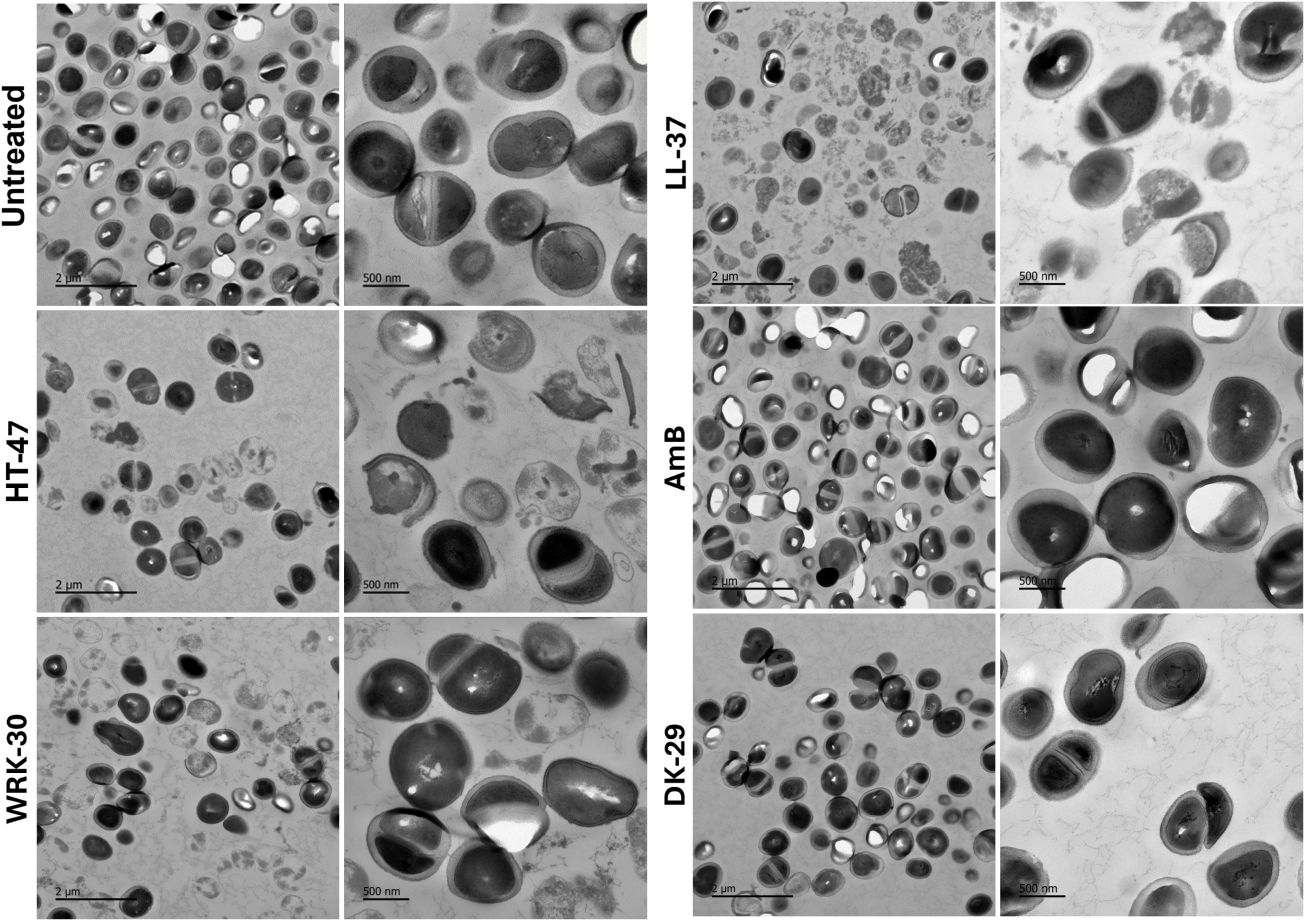
Transmission electron microscopy of *Candida auris* after peptide treatment. Representative TEM images of *C. auris* incubated overnight with CLEC3A-derived peptides (HT-47, WRK-30, DK-29), LL-37 amphotericin B (AmB), or left untreated. Images are shown at 5 µm, 2 µm, and 500 nm magnification. Untreated, DK-29 treated, and AmB treated cells display intact cell morphology and higher cell numbers, whereas HT-47, WRK-30, and LL-37 induce cell wall disruption, intracellular structural damage, and abundant extracellular debris, indicating peptide-mediated lysis and membrane destabilisation.

Transmission electron microscopy was performed to visualise the ultrastructural effects of CLEC3A-derived peptides on *C. auris*. Representative images at 5 µm, 2 µm, and 500 nm magnification are shown for each treatment condition. Untreated cells, as well as those treated with DK-29 or amphotericin B, largely maintained an intact cell wall and cytoplasmic organisation and displayed a comparatively higher number of intact cells. In contrast, treatment with HT-47, WRK-30, or LL-37 resulted in marked morphological alterations characterised by disrupted cell walls, cytoplasmic disorganisation, and prominent extracellular cellular debris. A reduced number of intact fungal cells was also visible in these groups, consistent with pronounced membrane and structural damage induced by the peptides (**Figure 5**).

## Discussion

CLEC3A-derived peptides are promising candidates for the search for novel antimicrobial agents, particularly given the increasing resistance observed in pathogens to commonly used antimicrobial drugs (Ventola, 2015; Santajit and Indrawattana, 2016; Lee et al., 2021). Previous studies have demonstrated that CLEC3A-derived peptides exhibit antimicrobial activity against both Gram-positive and Gram-negative bacteria, such as Staphylococcus aureus and Escherichia coli, without causing toxic effects on primary human chondrocytes (PHC) and murine fibroblasts (NIH3T3 cells) (Elezagic et al., 2019; Meinberger et al., 2023). The modifications, such as those made to HT-47 to create the peptide WRK-30, have significantly enhanced its antimicrobial potency through structural changes, including the addition of tryptophan and linker modifications that improved peptide flexibility and membrane interactions (Meinberger et al., 2023). Together, these findings establish CLEC3A-derived peptides as effective antimicrobial agents with the potential to overcome limitations of current antibiotic therapy.

Previous studies focused on the antibacterial activity of CLEC3A-derived peptides against selected bacterial species and demonstrated their biocompatibility and *in vivo* efficacy. The present study extends these findings by testing additional ESKAPE pathogens and providing the first systematic analysis of antifungal activity, thereby broadening the therapeutic scope of CLEC3A-derived peptides.

In the present study, CLEC3A-derived peptides HT-47, WRK-30, and DK-29, along with LL-37, as a positive control, were tested against clinical strains of K. pneumoniae and A. baumannii and the fungal clinical strains of C. albicans, C. neoformans, and *C.auris*. These pathogens pose significant challenges to the healthcare system due to their tendency to develop resistance to conventional treatments, consistent with the WHO´s recognition of them as priority pathogens for new drug development (WHO, 2017). *K. pneumoniae* and *A. baumannii* are part of the ESKAPE pathogens, which are leading to nosocomial infections, with rapidly emerging strains that can evade even last-resort antibiotics (Rice, 2010). Similarly, testing fungi such as C. albicans and C. neoformans is highly relevant, as current antifungal agents are increasingly limited and resistance to these therapies is increasing (Wiederhold, 2017; Geddes-McAlister and Shapiro, 2019). The persistence and severity of fungal biofilms, particularly in immunocompromised individuals, highlight the urgent need for new therapeutic approaches (Nobile and Johnson, 2015).

The viable count assays performed in this study showed that both HT-47 and WRK-30 significantly inhibited bacterial and fungal growth in a concentration-dependent manner. Regarding *K. pneumoniae* and HT-47, a significant reduction is observed at a concentration of 1.25 µM, indicating that the native peptide already possesses considerable antimicrobial potential. However, the assumption that the improved peptide WRK-30 exhibits even greater antibacterial activity due to its modifications cannot be demonstrated for K. pneumoniae, as WRK-30 shows only a significant decrease in bacterial growth at the higher concentration of 2.5 µM. Similar results are observed for A. baumannii, with a significant decrease in bacterial growth in HT-47 already evident at a concentration of 0.625 µM. These findings demonstrate that, although WRK-30, which was optimised for enhanced interactions with bacterial membranes (tryptophan residue), is highly potent against these bacterial pathogens, the less modified HT-47 is already highly potent at lower concentrations. The results of the study show a significant antibacterial activity of the CLEC3A-derived peptides Ht-47 and WRK-30 against both bacteria *K. pneumoniae* and *A. baumannii* at low micromolar concentrations. Although the enhanced peptide WRK-30 does not show a statistically significant improvement in bacterial killing compared with HT-47, the combined effects of its structural modifications, including reduced cytotoxicity, may make WRK-30 a more promising therapeutic candidate for bacterial control (Meinberger et al., 2023).

Fungal infections also represent an increasing global health challenge, driven by the rise of opportunistic and multidrug-resistant pathogens. Among these, *C. albicans, C. neoformans*, and *C. auris* are of particular clinical importance, as they are associated with severe systemic and device-related infections, especially in immunocompromised patients. The therapeutic management of these infections remains limited by the small number of available antifungal drug classes, their potential toxicity, and the emergence of resistant strains. Consequently, the development of novel antifungal agents is urgently needed (Liu et al., 2025).

We therefore investigated the antifungal activity of CLEC3A-derived peptides HT-47 and WRK-30 against *C. albicans, C. neoformans*, and *C. auris*. The viable count assays revealed that both peptides exhibit pronounced antifungal activity across all tested fungal species. Against *C. albicans*, HT-47 and WRK-30 achieved comparable reductions in fungal growth at the same concentration (1.25 µM), indicating similar efficacy to LL-37. These findings suggest that the antimicrobial mechanism of CLEC3A-derived peptides is not restricted to bacteria but also extends to fungal pathogens (Elezagic et al., 2019). In the case of *C. neoformans*, both peptides induced significant growth inhibition starting at 0.625 µM, with HT-47 showing slightly higher potency than WRK-30 at lower concentrations. The results obtained with *C. auris* are particularly relevant, given the pathogen’s increasing prevalence and multidrug resistance. Both HT-47 and WRK-30 exhibited potent antifungal activity, with highly significant reductions in fungal survival already at 0.625 µM. These findings highlight the potential of CLEC3A-derived peptides as promising antifungal candidates, particularly against *C. auris*, and further emphasise their broad-spectrum antimicrobial capacity. Comparison of MIC_50_ values revealed that the CLEC3A-derived peptides HT-47 and WRK-30 exhibit antimicrobial activity comparable to that of LL-37 across both bacterial and fungal species.

Previous studies have shown that CLEC3A-derived peptides exert their antibacterial activity primarily through membrane permeabilisation, a common mechanism of action among antimicrobial peptides. Using fluorescence microscopy, these peptides were observed to disrupt the membranes of both gram-positive and gram-negative bacteria, consistent with their cationic and amphipathic nature. Moreover, transmission electron microscopy demonstrated that CLEC3A-derived peptides induce pronounced structural alteration at the bacterial cell surfaces, resulting in compromised membrane integrity and increased permeability (Elezagic et al., 2019). While these findings have provided insights into the antibacterial mode of action, the mechanistic basis of their antifungal activity remains to be elucidated. Given that many amicrobial peptides, including LL-37, exert their effects by targeting and altering both the fungal cell wall and cell membrane, it was hypothesised that CLEC3A-derived peptides might employ a similar strategy (López-García et al., 2005; Tsai et al., 2011; Luo et al., 2019). To explore this possibility and provide visual evidence, scanning electron microscopy (SEM) and transmission electron microscopy (TEM) were applied to assess the morphological changes in the fungi following peptide treatment. The SEM data provide strong morphological evidence for the antifungal activity of CLEC3A-derived peptides against *C. albicans, C. neoformans*, and *C. auris*. Treatment with HT-47 and WRK-30 consistently resulted in extensive cellular disruption, comparable to that induced by the positive control peptide LL-37 and the antifungal drug Amphotericin B. The fibrillar structures and cellular remnants observed in the SEM images are characteristic of membrane damage and cell lysis, supporting the hypothesis that CLEC3A-derived peptides are not only active against bacteria but also, like other AMPs such as LL-37, exert potent antifungal effects by compromising fungal membrane integrity.

TEM analysis revealed pronounced structural damage in *C. auris* following treatment with the CLEC3A-derived peptides HT-47 and WRK-30, similar to the effects observed with LL-37. These alterations included loss of cell wall integrity, cytoplasmic disruption, and accumulation of extracellular debris, consistent with membrane-targeting activity. In contrast, untreated cells and those exposed to the inactive peptide DK-29 or amphotericin B largely retained intact morphology and increased in number, supporting the notion that CLEC3A-derived peptides induce direct cell disruption rather than merely inhibiting growth. Together, these ultrastructural observations corroborate our functional assays and provide visual evidence that membrane damage contributes to the antifungal activity of CLEC3A-derived peptides against *C. auris*.

The broad antibacterial and antifungal activity observed for CLEC3A-derived peptides highlights their therapeutic potential but also raises questions about their potential toxicity to host cells. Previous studies showed almost no toxicity against primary human chondrocytes and murine fibroblasts, consistent with the cartilage-associated origin of CLEC3A (Elezagic et al., 2019; Meinberger et al., 2023). However, broad-spectrum antimicrobial peptides primarily act through interactions with cellular membranes, a feature that can reduce selectivity and increase the likelihood of host-cell interactions. Well-characterised peptides such as LL-37 illustrate this, as their membrane-directed activity supports broad antimicrobial efficacy while also being associated with cytotoxic and hemolytic effects (Dürr et al., 2006; Buda De Cesare et al., 2020). A major factor in this lack of selectivity is hydrophobicity, which is essential for disrupting microbial membranes but often leads to collateral damage to mammalian cells (Aoki and Ueda, 2013; Meinberger et al., 2023). Given that fungal pathogens share aspects of membrane organisation with human cells, the broad-spectrum activity observed here warrants careful consideration in the context of host cell safety and translational applicability (Rex et al., 2001).

The morphological damage observed in peptide-treated fungal cells provides strong evidence for a membrane-associated mode of action; however, the precise molecular interactions underlying this effect remain undefined. While membrane disruption is a common mechanism among cationic antimicrobial peptides, multiple studies indicate that initial membrane destabilisation can promote further interactions with specific lipid components, such as sterols, or allow peptides to access intracellular targets (Hale and Hancock, 2007; Wenzel et al., 2014). In fungal cells, factors such as ergosterol content and lipid packing likely influence peptide activity and selectivity (Li et al., 2021). Accordingly, the membrane damage observed for CLEC3A-derived peptides may represent one component of a more complex mechanism shaped by differences in membrane composition across bacterial, fungal, and host cells, which warrants further investigation.

Biofilm formation is a major virulence and resistance mechanism of *C. auris*, contributing to its persistence on medical devices and tolerance to antifungal treatment. Given the limited efficacy of conventional antifungals against biofilm-associated *C. auris*, targeting biofilm formation is a crucial therapeutic strategy (Ahmad and Alfouzan, 2021; Czajka et al., 2023). Our findings demonstrate that the CLEC3A-derived peptides HT-47 and WRK-30 strongly inhibit biofilm formation by *C. auris* in a concentration-dependent manner, achieving up to 64% inhibition with WRK-30 at the highest tested concentration. The activity of WRK-30 was comparable to that of the host-defence peptide LL-37, which is already known to inhibit biofilm formation by other pathogens (Memariani and Memariani, 2023). Biofilm formation is a major virulence and persistence mechanism of C. auris, contributing to its remarkable resistance to antifungal therapy and disinfection. Biofilm-associated C. auris cells are particularly problematic in clinical environments, where they colonise medical devices and surfaces, leading to recurrent and difficult-to-treat infections (Ramage et al., 2006; Sherry et al., 2017; Horton and Nett, 2020). Consistent with this, previous *in vivo* work demonstrated that CLEC3A-derived peptides, HT-47 and WRK-30, when coated onto titanium implants and placed subcutaneously in a murine *S. aureus* infection model, significantly reduced the bacterial burden both on the implant surface and in the surrounding tissue. The biofilm-preventive effect in a clinically relevant biomaterial context highlights the capacity of these peptides to interfere with device-associated infections *in vivo* (Meinberger et al., 2025). Our current findings extend this concept to C. auris, demonstrating that the same peptides are also capable of inhibiting fungal biofilm formation. In contrast, amphotericin B, an established clinical antifungal, exhibited only limited activity against *C. auris* biofilms, achieving significant inhibition only at high concentrations. The observation aligns with previous reports describing reduced efficacy and resistance of C. auris to AmB (Lockhart et al., 2017; Escandón et al., 2019). The low activity of AmB in our assay underscores the clinical challenge posed by biofilm-associated C. auris infections, in which standard antifungals often fail to achieve sufficient efficacy.

In summary, CLEC3A-derived peptides HT-47 and WRK-30 exhibit strong bactericidal and fungicidal activity, inhibit C. auris biofilm formation, and induce ultrastructural damage consistent with membrane-targeting mechanisms, positioning them as promising candidates to address the urgent need for new antimicrobials against multidrug-resistant pathogens.

## Author’s note

CLEC3A-derived antimicrobial peptides (AMP) are covered by a pending German patent (DE 10 2018 113 988.8 A1) and an international patent application (PCT/EP2024/053920).

## Data Availability

The raw data supporting the conclusions of this article will be made available by the authors, without undue reservation.
